# Mosaic chromosomal alterations in blood across ancestries using whole-genome sequencing

**DOI:** 10.1038/s41588-023-01553-1

**Published:** 2023-10-30

**Authors:** Yasminka A. Jakubek, Ying Zhou, Adrienne Stilp, Jason Bacon, Justin W. Wong, Zuhal Ozcan, Donna Arnett, Kathleen Barnes, Joshua C. Bis, Eric Boerwinkle, Jennifer A. Brody, April P. Carson, Daniel I. Chasman, Jiawen Chen, Michael Cho, Matthew P. Conomos, Nancy Cox, Margaret F. Doyle, Myriam Fornage, Xiuqing Guo, Sharon L. R. Kardia, Joshua P. Lewis, Ruth J. F. Loos, Xiaolong Ma, Mitchell J. Machiela, Taralynn M. Mack, Rasika A. Mathias, Braxton D. Mitchell, Josyf C. Mychaleckyj, Kari North, Nathan Pankratz, Patricia A. Peyser, Michael H. Preuss, Bruce Psaty, Laura M. Raffield, Ramachandran S. Vasan, Susan Redline, Stephen S. Rich, Jerome I. Rotter, Edwin K. Silverman, Jennifer A. Smith, Aaron P. Smith, Margaret Taub, Kent D. Taylor, Jeong Yun, Yun Li, Pinkal Desai, Alexander G. Bick, Alexander P. Reiner, Paul Scheet, Paul L. Auer

**Affiliations:** 1 Department of Internal Medicine, University of Kentucky, Lexington, KY USA; 2 Public Health Sciences Division, Fred Hutchinson Cancer Center, Seattle, WA USA; 3 Department of Biostatistics, University of Washington, Seattle, WA USA; 4 Department of Computer Science, Department of Biological Sciences, University of Wisconsin-Milwaukee, Milwaukee, WI USA; 5 Department of Epidemiology, University of Texas M.D. Anderson Cancer Center, Houston, TX USA; 6 University of South Carolina, Columbia, SC USA; 7 Division of Biomedical Informatics and Personalized Medicine, School of Medicine University of Colorado Anschutz Medical Campus, Aurora, CO USA; 8 Cardiovascular Health Research Unit, Department of Medicine, University of Washington, Seattle, WA USA; 9 Human Genetics Center, Department of Epidemiology, Human Genetics, and Environmental Sciences, School of Public Health, The University of Texas Health Science Center at Houston, Houston, TX USA; 10 Cardiovascular Health Research Unit, Department of Medicine, University of Washington Seattle, Seattle, WA USA; 11 Department of Medicine, University of Mississippi Medical Center, Jackson, MS USA; 12 Brigham and Women’s Hospital, Boston, MA USA; 13 Department of Biostatistics, University of North Carolina at Chapel Hill, Chapel Hill, NC USA; 14 Channing Division of Network Medicine, Brigham and Women’s Hospital, Boston, MA USA; 15 Division of Genetic Medicine, Vanderbilt University Medical Center, Nashville, TN USA; 16 Department of Pathology and Laboratory Medicine, The University of Vermont Larner College of Medicine, Colchester, VT USA; 17 University of Texas Health Science Center at Houston, Houston, TX USA; 18 The Institute for Translational Genomics and Population Sciences, Department of Pediatrics, The Lundquist Institute for Biomedical Innovation at Harbor-UCLA Medical Center, Torrance, CA USA; 19 Department of Epidemiology, School of Public Health, University of Michigan, Ann Arbor, MI USA; 20 Department of Medicine, University of Maryland Baltimore, Baltimore, MD USA; 21 The Charles Bronfman Institute for Personalized Medicine, Icahn School of Medicine at Mount Sinai, New York, NY USA; 22 Novo Nordisk Foundation Center for Basic Metabolic Research, Faculty of Health and Medical Sciences, University of Copenhagen, Copenhagen, Denmark; 23 Division of Biostatistics, Medical College of Wisconsin, Milwaukee, WI USA; 24 National Institutes of Health, Bethesda, MD USA; 25 Division of Allergy and Clinical Immunology, Department of Medicine, Johns Hopkins University School of Medicine, Baltimore, MA USA; 26 Center for Public Health Genomics, Department of Public Health Sciences, University of Virginia School of Medicine, Charlottesville, VA USA; 27 Department of Epidemiology, University of North Carolina Chapel-Hill, Chapel Hill, NC USA; 28 Department of Laboratory Medicine and Pathology, University of Minnesota Medical School, Minneapolis, MN USA; 29 Cardiovascular Health Research Unit, Department of Medicine, Department of Epidemiology, Department of Health Systems and Population Health, University of Washington, Seattle, WA USA; 30 Department of Genetics, University of North Carolina at Chapel Hill, Chapel Hill, NC USA; 31 Department of Epidemiology, Boston University, Boston, MA USA; 32 Division of Sleep Medicine, Harvard Medical School, Boston, MA USA; 33 Institute for Social Research, Survey Research Center, University of Michigan, Ann Arbor, MI USA; 34 Institute for Biomedical Informatics, University of Kentucky, Lexington, KY USA; 35 Department of Biostatistics, Bloomberg School of Public Health, Johns Hopkins University, Baltimore, MA USA; 36 Department of Biostatistics, Department of Genetics, Department of Computer Science, University of North Carolina Chapel-Hill, Chapel Hill, NC USA; 37 Department of Medicine, Weill Cornell Medicine, New York, NY USA; 38 Department of Epidemiology, University of Washington, Seattle, WA USA; 39 Division of Biostatistics, Institute for Health and Equity, and Cancer Center, Medical College of Wisconsin, Milwaukee, WI USA

**Keywords:** Genetics, Cancer

## Abstract

Megabase-scale mosaic chromosomal alterations (mCAs) in blood are prognostic markers for a host of human diseases. Here, to gain a better understanding of mCA rates in genetically diverse populations, we analyzed whole-genome sequencing data from 67,390 individuals from the National Heart, Lung, and Blood Institute Trans-Omics for Precision Medicine program. We observed higher sensitivity with whole-genome sequencing data, compared with array-based data, in uncovering mCAs at low mutant cell fractions and found that individuals of European ancestry have the highest rates of autosomal mCAs and the lowest rates of chromosome X mCAs, compared with individuals of African or Hispanic ancestry. Although further studies in diverse populations will be needed to replicate our findings, we report three loci associated with loss of chromosome X, associations between autosomal mCAs and rare variants in *DCPS*, *ADM17*, *PPP1R16B* and *TET2* and ancestry-specific variants in *ATM* and *MPL* with mCAs in *cis*.

## Abstract (web summary)

A method that allows for the detection of mosaic chromosomal alterations from blood whole-genome sequencing data highlights ancestry-specific differences in the distribution of common and rare germline susceptibility variants.

## Main

Mosaicism refers to the presence of genetically distinct lineages of cells resulting from a single zygote in a multicellular organism. The clone with a somatic mutation may comprise a substantial fraction of cells in a tissue, which have risen to detectable frequency due to selective advantage or drift. Surveys of blood samples from healthy donors have revealed extensive age-related clonal mosaicism, which can involve somatic mutations ranging in size from a single nucleotide to large typically megabase-scale alterations, which include chromosomal losses, gains and copy neutral loss of heterozygosity (CN-LOH) that are >1–2 Mb and are referred to as mosaic chromosomal alterations (mCAs)^1–7^. The presence of these acquired mutations in autosomes are more common in men and confer an approximately tenfold higher risk for the development of hematological malignancies in otherwise healthy adults^3,4,8,9^.

Mosaicism in blood of single nucleotide variants (SNVs) for acquired leukemogenic mutations in individuals without evidence of hematologic malignancy, dysplasia or cytopenia is known as clonal hematopoiesis of indeterminate potential (CHIP). Studies of mosaic SNVs have shown an ~13-fold higher risk for development of hematological malignancies in those with CHIP mutations^10–12^. Beyond cancer, mosaicism in blood has been associated with other chronic diseases, further highlighting its potential as a biomarker with clinical utility^11,13–15^.

The largest studies of mCAs have surveyed DNA array data from individuals of European (EA) or Japanese ancestries^6,16^. Yet, the landscape of mCAs in more genetically diverse populations and from whole-genome sequencing (WGS) remains underexplored. In this Article, to address this void we investigated mCAs using WGS data from the Trans-Omics for Precision Medicine (TOPMed) program in 67,390 individuals, including 20,132 individuals of African American ancestry (AA), 7,608 individuals of Hispanic ancestry (HA), and 1,203 individuals of East Asian (EAS) ancestry. We demonstrate for the first time the use of a haplotype-based methodology for the detection of mCAs from high-coverage (30×) WGS data. This methodology allowed for detection of mCAs at mutant cell fractions below 1% and enabled association analyses of both rare and common germline variation with the presence of mCAs.

## Results

### Genomic landscape of mCAs

**Fig. 1:**
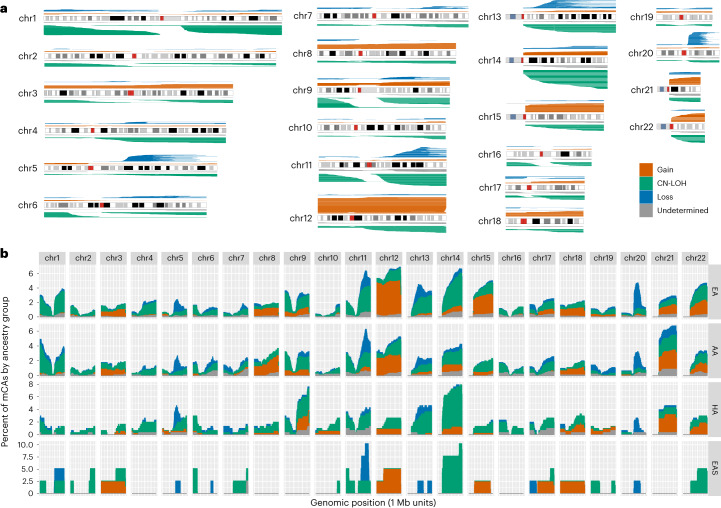
Genomic distribution of autosomal mCAs. **a**, mCA calls across autosomal chromosomes. **b**, Histogram of mCA calls across the genome for each genetic ancestry group. The *X* axis is shown in 1 Mb windows for each chromosome and the *Y* axis is the percent of mCA calls for a given genetic ancestry group that span the genomic window.

**Fig. 2:**
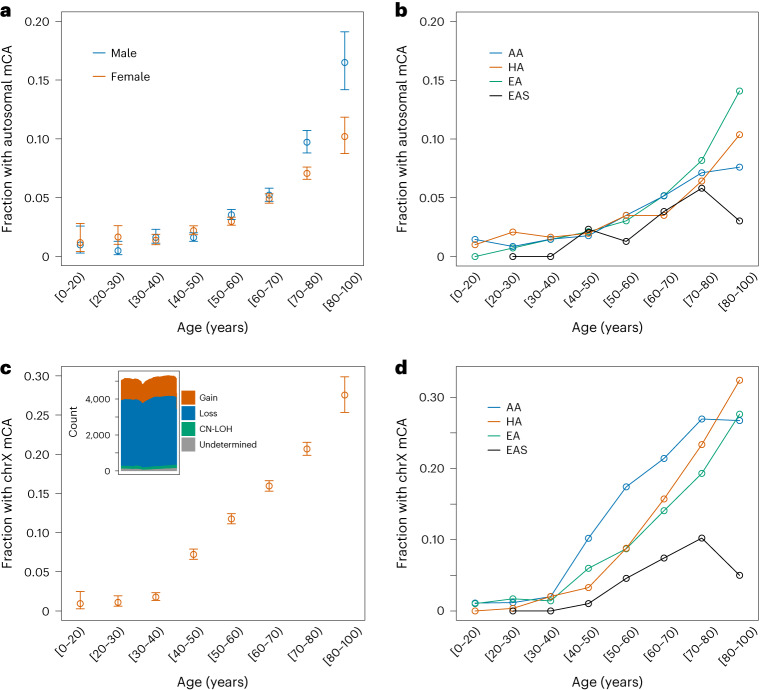
Rate of mCAs by age. **a**, Fraction of females (*n* = 41,895 biologically independent individuals) and males (*n* = 25,495 biologically independent individuals) with one or more autosomal mCA across age bins. Error bars represent 95% confidence intervals. **b**, Fraction of individuals across different genetic ancestry groups with one or more autosomal mCA across age bins. **c**, Fraction of females (*n* = 41,895 biologically independent individuals) with a chrX mCA across age bins. Error bars represent 95% confidence intervals. The inset shows a histogram of chrX mCA calls. The *X* axis shows 1 Mb windows across chrX, and the *Y* axis is the number of mCA calls that span the genomic window. **d**, Fraction of females across different genetic ancestry groups with a chrX mCA across age bins.

We detected 3,659 autosomal mCAs in 67,390 TOPMed samples (Fig. 1 and Supplementary Table 1). A total of 3,017 samples (4.47%) had at least one detectable autosomal mCA, of which 414 had mCAs in more than one autosomal chromosome. As reported previously, the rate of mCAs increased with age (Fig. 2a), and the rate of autosomal mCAs for males was higher than that for females (odds ratio (OR) 1.19, *P* = 6.3 × 10^−5^, Fig. 2a)^1–5,7^. To investigate the accuracy of our mCA calls, we compared the mCA detection in our WGS data with array-based mCA calls on a subset of 18,093 individuals from four different cohorts (Multi-Ethnic Study of Atherosclerosis (MESA), Cardiovascular Health Study (CHS), Women’s Health Initiative (WHI) and the Genetic Epidemiology of Chronic Obstructive Pulmonary Disease (COPDGene), see Supplementary Note 1) that were typed on five different arrays. Overall, we found that mCA detection was more sensitive with the TOPMed WGS data, particularly for mCAs with clonal fractions (CFs) below 5%. The array-based mCA calls were highly concordant with the TOPMed WGS-based calls for high CF >10% events with 82% of WGS-based autosomal mCAs called by array. CN-LOH calls had higher concordance rates than either gains or losses (Supplementary Note 1).

Autosomal mCAs were categorized as gain, loss, CN-LOH or undetermined. The most frequent autosomal mCAs (*n* > 100) were 14q CN-LOH, 12p and 12q gains, 20q loss, 11q CN-LOH and 1p CN-LOH (Fig. 1 and Supplementary Table 2). We tested for differences in the rates of each autosomal mCA across chromosome arms in males and females. We found significant enrichment in males for chromosome 20q arm loss (OR 2.76, *P* = 1.1 × 10^−5^) and 15q gain (OR 2.73, *P* = 2.1 × 10^−3^). Multiple other loci exhibiting mCAs had a significant sex-specific enrichment with all having higher rates in males (Supplementary Table 3).

**Extended Data Fig. 1:**
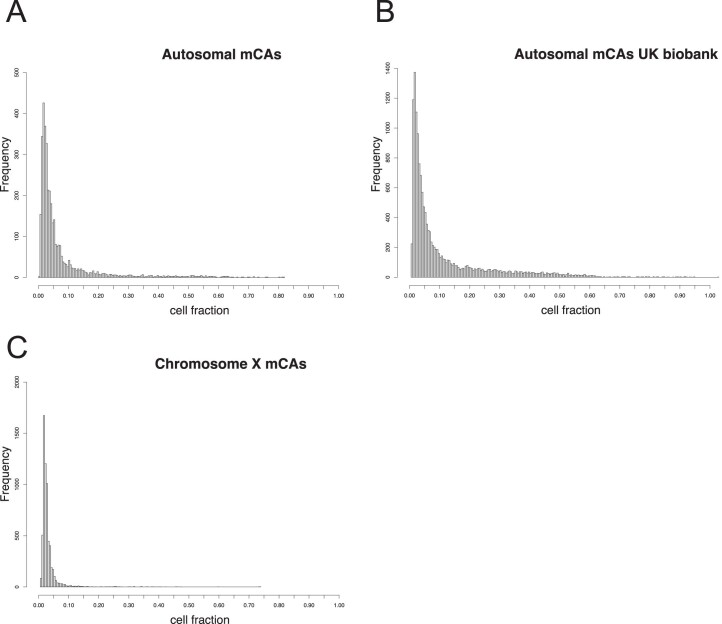
Distribution of the estimated mutant cell fraction of mosaic chromosomal alterations (mCAs). **a**., frequency of mutant cell fractions for autosomal mCAs called in TOPMed. **b**., frequency of mutant cell fractions for autosomal mCAs called in the UKBiobank. **c**., frequency of mutant cell fractions for chromosome X mCAs called in TOPMed.

**Extended Data Fig. 2:**
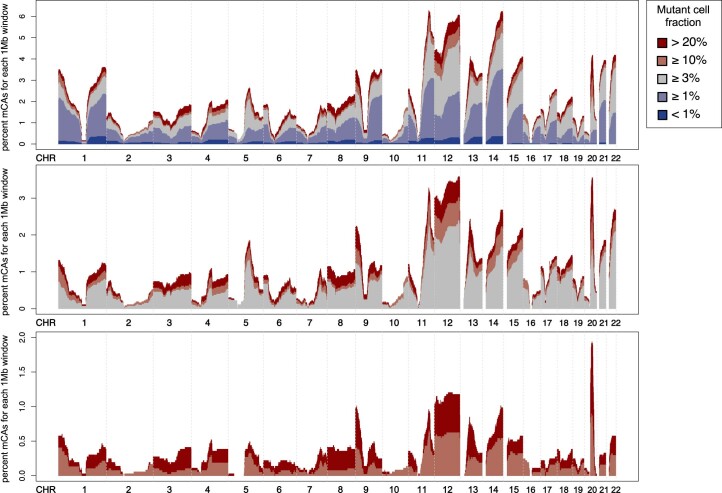
Genomic distribution of mosaic chromosomal alterations (mCAs) across mutant cell fraction (CF) categories. The percent of mCAs (number of mCA calls spanning genomic location / all mCA calls) is shown on the vertical axis and genomic location is shown on the horizontal axis. Bins span 1megabase (Mb) and mCAs with CF>20% are in dark red, CF$\ge$10% in light red, CF$\ge$3% in grey, CF$\ge$1% in light blue, and CF<1% in dark blue. CHR; chromosome.

The majority (82%) of detectable autosomal mCAs were estimated to be present at cell fractions less than 10%, with a large proportion (44%) present at estimated cell fractions less than 3% (Extended Data Fig. 1). Only 8.5% of autosomal mCAs were present at estimated cell fractions greater than 20%. When restricting to mCAs present at cell fractions of 10% or greater, chromosome 20 mCAs were most frequent (~2% of all autosomal mCAs with CF >10%), followed by mCAs on chromosome 12 (~1% of all autosomal mCAs with CF >10%). This distribution contrasts with the distribution of autosomal mCAs across all cell fractions, where the most frequently altered chromosomes are 11, 12 and 14 (Extended Data Fig. 2). This variation suggests differential fitness advantages across clones with different mCAs.

**Extended Data Fig. 3:**
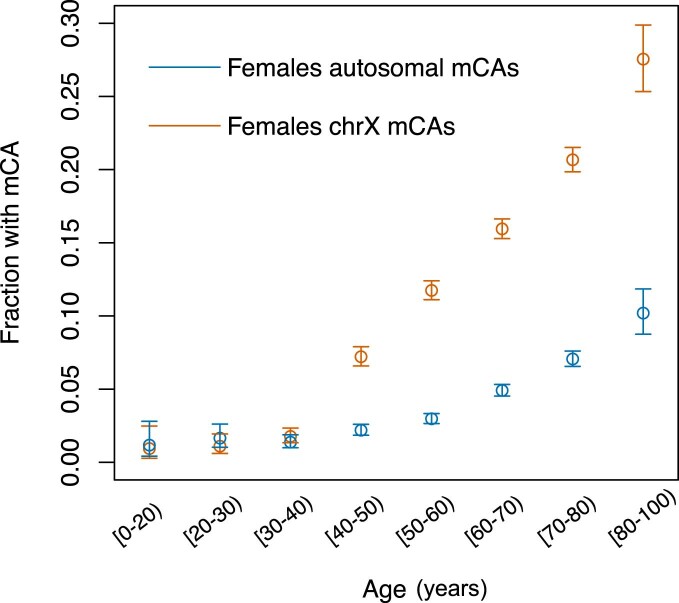
Rate of mosaic chromosomal alterations (mCAs) by age in females in TOPMed. Age bins in years are plotted on the horizontal axis and the fraction of females carrying an mCA is plotted on the vertical axis. Autosomal mCAs are shown in blue, and chromosome X (chrX) mCAs are shown in red. Error bars represent 95% confidence intervals. There were n = 41,895 biologically independent individuals included in this analysis.

**Extended Data Fig. 4:**
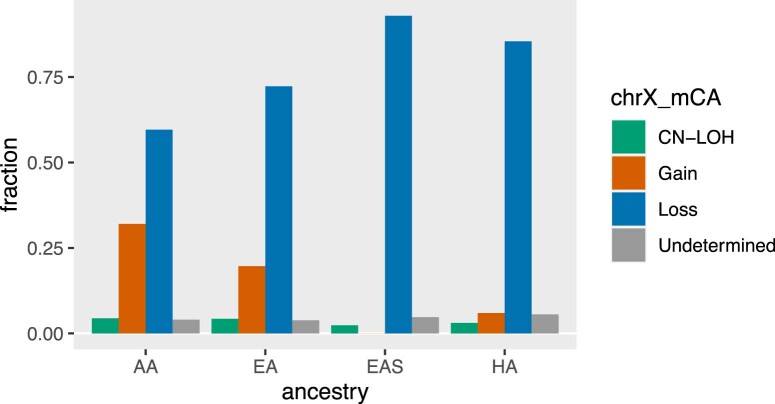
Rates of chromosome X mosaic chromosomal alterations (mCAs) by genetic ancestry. The fraction of chromosome X (chrX) mCAs is plotted by ancestry and type of mCA with copy-neutral loss of heterozygosity (CN-LOH) in green, Gain in orange, Loss in blue, and Undetermined in grey. AA; African American ancestry, HA; Hispanic ancestry, EA; European ancestry, EAS; East Asian ancestry.

Using the same methodology as for autosomal mCAs, we surveyed mCAs on chromosome X (chrX) from 41,895 female samples in the TOPMed cohort and identified 6,207 mCAs. The rate of chrX mCAs was significantly higher (*P* = 2.2 × 10^−16^) than for autosomal mCAs with 13.7% of females harboring an mCA on chrX in contrast to 4.38% with mCAs on the autosomes (Extended Data Fig. 3). Overall mCAs on chrX had lower estimated cell fraction than mCAs on the autosomes (Extended Data Fig. 1). Most of the mCAs on chrX (68.8%) were losses (Fig. 2c and Extended Data Fig. 4). The presence of an mCA on chrX showed a positive association with autosomal mCAs (OR 1.21, *P* = 0.003).

### Distribution of mCAs across ancestries

TOPMed samples have been previously categorized into five genetic ancestry groups, namely AA, HA, EA, EAS and a fifth set of individuals for which genetic ancestry could not be confidently determined (Methods and Supplementary Table 1). We sought to make comparisons of mCA rates across these ancestry groups. Because haplotype-based detection of mCAs is made possible by analysis of signal at heterozygous genotypes, differences in heterozygosity levels across groups could drive differences in sensitivity for detection of mCAs (Supplementary Table 4). To control for the impact of variable heterozygosity rates, we downsampled heterozygous sites in the AA, HA and EA groups to match the distribution in the EAS group, which has the lowest heterozygosity rate, and re-ran the mCA detection procedure (Methods). We contrasted the call sets before and after the downsampling procedure, which resulted in fewer mCA calls in the AA, HA and EA groups and the largest impact was observed for mCAs in the undetermined category (Supplementary Tables 5 and 6). We ran analyses contrasting mCA rates across ancestry groups using the mCA call set both before and after downsampling and observed differences across groups which were consistent in direction and statistically significant using the mCA call set both before and after downsampling. In the following, we present results for the downsampled data as it more accurately estimates differences between ancestry groups.

**Extended Data Fig. 5:**
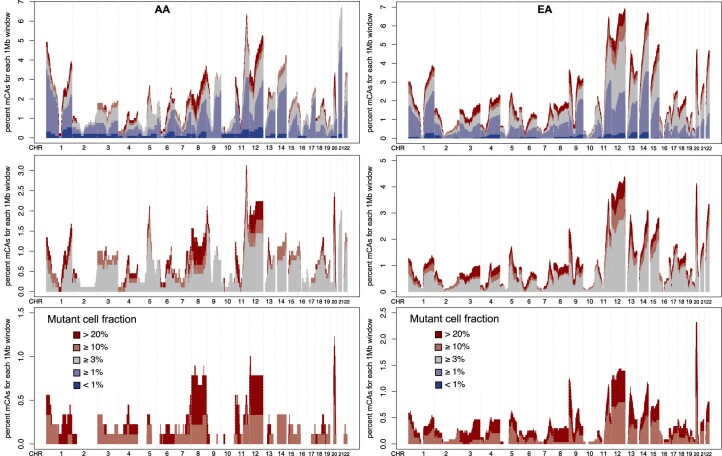
Genomic distribution of mosaic chromosomal alterations (mCAs) across mutant cell fraction (CF) categories in. African American ancestry **(AA) and** European ancestry **(EA) groups**. The percent of mCAs (number of mCA calls spanning genomic location / all mCA calls) is shown on the vertical axis and genomic location is shown on the horizontal axis. Bins span 1 megabase (Mb) and mCAs with CF>20% are in dark red, CF ≥ 10% in light red, CF ≥ 3% in grey, CF ≥ 1% in light blue, and CF<1% in dark blue. The left panel displays mCAs detected in AAs and the right panel displays mCAs detected in EAs.

The AA, HA and EAS groups exhibited significantly lower autosomal mCA rates relative to EA (OR 0.76, *P* = 1.2 × 10^−5^ for AA; OR 0.71, *P* = 0.0009 for HA; and OR 0.62, *P* = 0.007 for EAS). Next, we contrasted the autosomal regions that harbored mCAs between AA and EA, the two genetic ancestry groups with the largest sample size and thus most amenable to such a comparison (Fig. 1b and Extended Data Fig. 5). In the EA group, autosomal mCAs were observed most frequently on chromosomes 11, 12 and 14. For the AA group, the most frequent autosomal mCAs were observed on chromosomes 11, 12 and 21. When mCAs at cell fractions less than 3% were excluded, the most frequent autosomal mCAs for the AA and EA ancestry groups were observed on chromosome 11, 12 and 20 (Extended Data Fig. 5). We formally tested for differences across ancestry groups for the ten most frequent mCA types found in AA, EA and HA adjusting for age, age^2^, sex and study (Supplementary Tables 7 and 8). We observed that chromosome 12q gains, 14q CN-LOH and 20q loss were more frequent in the EA group relative to AA and HA (*P* < 0.05). The rate of mCAs on chromosome 13q was higher for the EA group compared with the AA group (*P* = 0.016). Of note, chromosome alterations on 13q and gains of chromosome 12 are associated with chronic lymphocytic leukemia (CLL), which has a higher incidence in EA compared with other ancestry groups^17,18^. We observed that 8q gains are more common in the AA group compared with EA (*P* = 0.036), a difference that has also been observed in breast, prostate, endometrial and ovarian cancers suggestive of shared drivers of 8q amplifications across tissues^19^. The observed ancestry-specific associations for mCA subtypes could be driven by germline genetic variation and/or environmental exposures that differ across these ancestry groups. To investigate the potential contribution of genetic drivers, we tested for associations between estimated continental African genetic ancestry at the chromosome level with mCAs in the AA group (Methods). The direction of the association with estimated African ancestry was consistent with the results above for 8q gains, 13q CN-LOH, 14q CN-LOH and 20q loss, but not for chromosome 12 gains (Supplementary Table 9).

We observed a higher chrX mCA rate in AA and HA compared with EA (OR 1.67, *P* = 2.5 × 10^−33^ for AA and OR 1.36, *P* = 0.00013 for HA). The chrX mCA rate for EAS was lower compared with EA (OR 0.49, *P* = 3.2 × 10^−5^). To determine if the association was driven by a specific type of mCA, we tested for associations between genetic ancestry and chrX loss and gain separately. ChrX loss rates were higher in AA (OR 1.59, *P* = 2.58 × 10^−20^) and in HA (OR 1.42, *P* = 2.13 × 10^−5^) compared with EA ancestry groups. As with autosomal mCAs, chrX loss was lower in EAS (OR 0.41, *P* = 3.5 × 10^−7^) compared with EA and the AA group demonstrated a higher rate of chrX gains compared with the EA group (OR 2.06, *P* = 6.27 × 10^−22^). For individuals in the AA group we tested for an association between chrX mCAs and estimated proportion of African ancestry on chrX and observed a lower rate (*P* < 0.05) of chrX mCAs in individuals with less than 25% African ancestry on chrX compared with those in the top three quartiles of African ancestry on chrX (OR 1.16–1.34) (for details, see Methods).

### Germline predictors of chromosomal alterations

We performed a WGS-based genome-wide association analysis (GWAS) between germline variants observed in TOPMed and presence of an mCA, separately for autosomal (*N* = 67,518) and chrX (*N* = 41,864) mCAs (Methods). Of the 30 variants reported to be associated with presence of an autosomal mCA in Loh et al.^6^, we replicated eight associations in the *TERT* gene locus (Supplementary Table 10) at a nominal significance level. No single variant was significant at a genome-wide Bonferroni-corrected *P*-value threshold (5 × 10^−8^) for the GWAS of autosomal mCAs. We also conducted a GWAS for loss-of-chrX (LoX) in females and found three genome-wide significant loci (Supplementary Table 11). The most significant association was with rs4973315 (OR 0.77, *P* = 4.74 × 10^−11^), a single nucleotide polymorphism (SNP) near the *SP140L* gene. A variant near *HLA-B* (rs9266255) was likewise associated with LoX (OR 1.17, *P* = 8.43 × 10^−10^). And an ancestry-differentiated variant (rs58502248; AA minor allele frequency (MAF) 0.07, EA MAF 0.002) was associated with LoX (OR 0.59, *P* = 1.31 × 10^−8^). Given our limited sample size (that is, small numbers of mCAs) for detecting genome-wide significant signals, these results suggest that LoX is under genetic control, a portion of which may be due to ancestry stratified variants such as rs58502248.

**Fig. 3:**
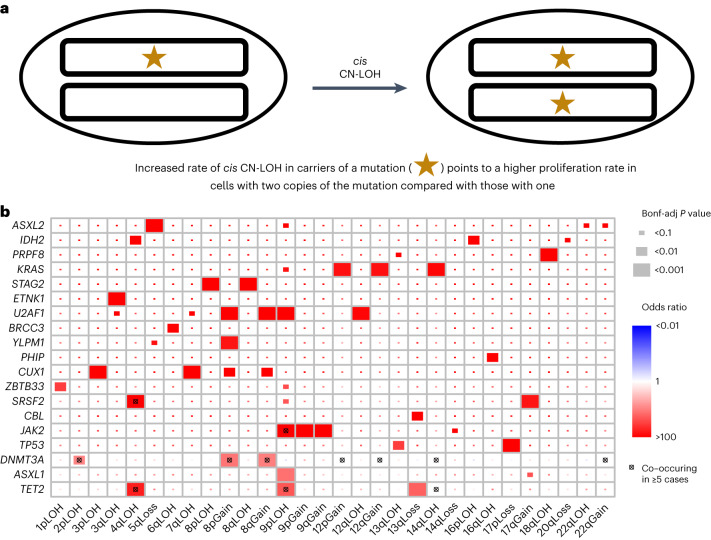
Co-occurrence of mCAs and CHIP mutations. **a**, Schematic of how a CHIP mutation may coincide with a CN-LOH event, leading to a proliferative advantage. **b**, Co-occurrence of CHIP and mCA mutations in 30 CHIP genes and 67 mCA events. Bonf-adj, Bonferroni adjusted.

Next, we performed *cis* analyses (Methods), testing for association between presence of an mCA and germline variants on the same chromosome arm as the mCA (Fig. 3a). Based on frequency in our dataset and importance from the literature, we defined the following mCA binary phenotypes: CN-LOH at *MPL*, CN-LOH at *ATM*, 11q CN-LOH, 1p CN-LOH, 12p gain, 12q gain, 14q CN-LOH and 20q loss (Supplementary Table 12). We found a 3′ untranslated region variant at the *ATM* gene (rs3092836) that was significantly associated with mCA at *ATM* (OR 26.41, *P* = 0.0013) and multiple other variants in *ATM* and *MPL* that were associated at a nominal (*P* < 0.05) level (Supplementary Table 13). Of the variants with a nominal association with CN-LOH at *ATM* and *MPL*, several varied by ancestry with some having MAF greater than 5% in AA but less than 0.1% in EA. Several variants present at minor allele count (MAC) <20 were estimated to have a large effect in *cis*-association analyses of CN-LOH at *ATM* and *MPL* as well, (Supplementary Table 14). One of these variants included rs56009889 at the *ATM* locus (OR 92, *P* = 2.5 × 10^−8^, MAC 7) which is associated with lung cancer risk^20^. We also replicated a splice donor variant (rs146249964, OR 296, *P* = 9.5 × 10^−8^, MAC 7) that was previously reported to be associated with CN-LOH of *MPL*^6^. The role of rs146249964 in hematopoiesis comes from clinical reports in individuals with congenital amegakaryocytic thrombocytopenia^21,22^. To determine whether these variants were selectively located on the haplotype that was duplicated in the CN-LOH events at *MPL* and *ATM*, we conducted an allelic shift analysis as in Loh et al. for variants with OR >1 and *P* < 0.05 from the *cis*-association analyses. We confirm the finding by Loh et al. at rs146249964 in *MPL*, where CN-LOH replaces the putatively deleterious rare allele for the common allele (*P* = 0.016, Supplementary Table 15). All six *ATM* variants tested showed a shift consistent with CN-LOH replacing the reference allele with the putatively deleterious allele (Supplementary Table 15). All but one *ATM* variant had the highest frequency in the AA group, including the rs3092836 variant, which is present at an estimated MAF of 8% in AA, 2% in EAS and HA, and 0.06% in EA.

To further investigate rare germline variants for association with autosomal and chrX mCAs, we implemented gene-centric aggregate rare variants tests for all variants with MAF <1% (Methods). We found 18 statistically significant associations between a burden of rare variants and presence of mCAs (Table 1, Supplementary Note 2 and Supplementary Dataset 1). The majority of the associations were driven by variants in *ATM* or *MPL*. Of these 18 associations, 11 were at or near *ATM* or *MPL* and were associated with CN-LOH at *MPL* (or 1p CN-LOH event) or with CN-LOH at *ATM* (or 11q CN-LOH event). The remaining signals were located at *DCPS* (with any autosomal mCA), *ADAM17* (with any autosomal mCA), *PPP1R16B* (with any autosomal mCA), *TET2* (with any autosomal mCA) and *OR4C16* (with chrX mCA).

**Table 1:** Associations between burden of rare variants and mCAs

Outcome	Variant grouping strategy	Gene	Number of variants	cMAC^a^	Odds ratio	*P* value
Autosomal mCA	coding_filter1	*MPL*	71	505	1.05	1.40 × 10^−7^
Autosomal mCA	coding_filter1	*DCPS*	80	626	1.04	4.80 × 10^−7^
Autosomal mCA	coding_filter1	*ADAM17*	33	72	1.12	1.00 × 10^−6^
Autosomal mCA	coding_filter1	*PPP1R16B*	14	27	1.22	9.60 × 10^−7^
Autosomal mCA	coding_filter1	*TET2*	163	171	1.06	1.50 × 10^−8^
Autosomal mCA	coding_noncoding_filter1	*MPL*	71	505	1.05	1.40 × 10^−7^
Autosomal mCA	coding_noncoding_filter1	*DCPS*	82	630	1.04	6.30 × 10^−7^
Autosomal mCA	coding_noncoding_filter1	*PPP1R16B*	14	27	1.22	9.60 × 10^−7^
chrX mCA	coding_filter1	*OR4C16*	21	174	1.04	2.40 × 10^−6^
11q CN-LOH	coding_filter1	*ATM*	23	30	1.17	9.90 × 10^−12^
11q CN-LOH	coding_noncoding_filter1	*ATM*	23	30	1.17	9.90 × 10^−12^
11q CN-LOH	coding_noncoding_filter1	*AP003392.2*	62	127	1.06	8.30 × 10^−5^
1p CN-LOH	coding_filter1	*MPL*	15	30	1.33	1.20 × 10^−32^
1p CN-LOH	coding_noncoding_filter1	*MPL*	15	30	1.33	1.20 × 10^−32^
*ATM* CN-LOH	coding_filter1	*ATM*	22	29	1.17	1.30 × 10^−10^
*ATM* CN-LOH	coding_noncoding_filter1	*ATM*	22	29	1.17	1.30 × 10^−10^
*MPL* CN-LOH	coding_filter1	*MPL*	15	28	1.33	5.40 × 10^−31^
*MPL* CN-LOH	coding_noncoding_filter1	*MPL*	15	28	1.33	5.40 × 10^−31^

^a^Cumulative minor allele count.

### Co-occurence of mCAs and CHIP mutations

**Extended Data Fig. 6:**
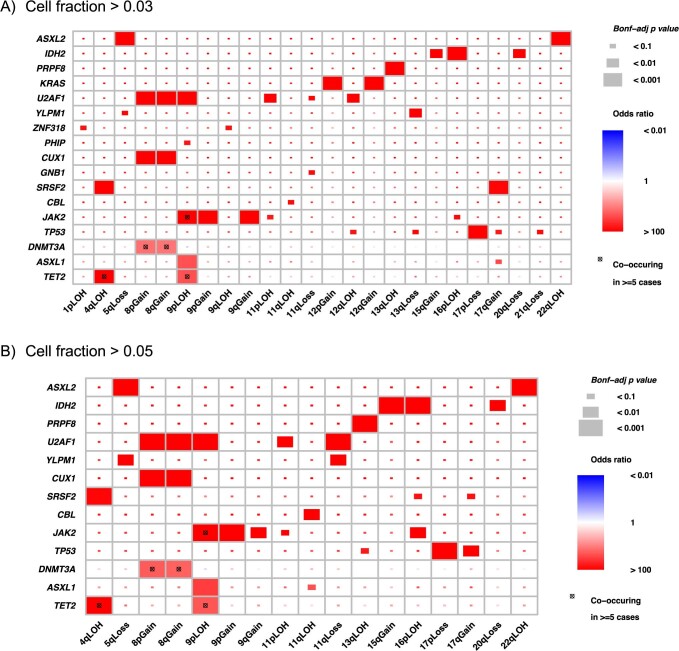
Co-occurrence of mosaic chromosomal alterations (mCAs) and clonal hematopoiesis of indeterminate potential (CHIP) mutations. Co-occurrence of CHIP and mCA mutations in 30 CHIP genes and 67 mCA events. **a**., co-occurrence of mCAs with CF > 0.03. **b**., co-occurrence of mCAs with CF > 0.05. All tests were two-sided z-tests and p-values were adjusted for multiple testing using the Bonferroni correction. Bonf-adj; Bonferroni-adjusted, LOH; loss of heterozygosity.

We interrogated the link between large structural alterations and single nucleotide mutations by tracking the co-occurence of somatic mutations in known CHIP genes that were mentioned in Bick et al. 2020 (*N* = 3,823) and mCAs (*N* = 8,402)^1^. Overall, individuals with CHIP were more likely to also carry an autosomal mCA (OR 2.76) or an mCA on chrX (OR 1.38, Supplementary Table 16). We observed ‘two hits’ at a number of cancer-associated genes (Fig. 3b). These included CHIP mutations co-occurring with CN-LOH at *TET2* (4q), *DNMT3A* (2p), *JAK2* (9p) and *CUX1* (7q). For *TP53*, we observe significant co-occurrence of somatic mutations with loss of chromosome arm 17p where *TP53* is located. Additionally, we observe significant co-occurence of somatic mutations in *SRSF2* and *KRAS* with gains of these genes. We make a similar observation for the *IDH2* oncogene (15q gain and *IDH2* somatic mutation); however, it is marginally significant. Loss of 13q, a common CLL chromosomal alteration, showed significant co-occurrence with somatic mutations in *TET2* and *CBL*^17^. Chromosome 8 gains displayed significant co-occurrence with somatic mutations in *DNMT3A*, *CUX1* and *U2AF1*. We repeated these analyses excluding mCAs with estimated mCA mutant cell fractions lower than 3%, or lower than 5% (Extended Data Fig. 6). At these higher mutant cell fractions, CHIP mutations in *DNMT3A* and *CUX1* did not exhibit significant co-occurrence with CN-LOH in *cis*, while CHIP mutations in *KRAS* did show significant co-occurrence with mCAs at the 3%, but not the 5%, mutant cell fraction threshold. Associations for *TET2*, *JAK2*, *TP53* and *SRSF2* mutations with *cis*-mCAs were still significant, as were associations of *DNMT3A*, *CUX1* and *U2AF1* mutations with chromosome 8 gains.

### Association between mCAs and hematologic traits and cancers

As has been previously reported, presence of autosomal mCAs at high CFs increases the risk of blood cancers greater than tenfold^4^. We investigated the association between autosomal mCAs, chrX mCAs and mCAs at either high (≥3%) or low CF (<3%) with both myeloid (52 cases and 7,691 controls) and lymphoid (215 cases and 7,291 controls) malignancies (Table 2). Autosomal mCAs were associated with an increased risk for lymphoid cancers (OR 2.94, *P* = 4.73 × 10^−7^) with a stronger effect for high CF mCAs (OR 3.78, *P* = 1.46 × 10^−7^). There were no associations between chrX mCAs with lymphoid cancers. Similar to the analysis in Niroula et al.^21,23^, we found an even stronger association with lymphoid cancers when we only considered autosomal mCAs that were classified as ‘lymphoid’ (OR 5.64, *P* = 6.60 × 10^−9^). For myeloid cancers, we found stronger associations with autosomal (OR 5.42, *P* = 1.35 × 10^−5^) and high CF mCAs (OR 7.77, *P* = 1.74 × 10^−6^) and no associations with low CF or chrX mCAs. To assess the possibility that these associations were implicating mCAs as biomarkers for early, subclinical disease, we re-ran the associations excluding individuals with cytopenias or cytoses (Methods). The associations with lymphoid cancers were attenuated with these exclusions in place, but the associations with myeloid cancers remained (Supplementary Table 17).

**Table 2:** Associations between myeloid and lymphoid malignancies and presence/absence of mcAs

Disease	Variable	OR	*P* value	*n* cases	*n* controls
Lymphoid	Autosomal mCA	2.94	4.73 × 10^−7^	215	7,691
Lymphoid	High CF^a^ autosomal mCA	3.78	1.46 × 10^−7^	215	7,691
Lymphoid	Low CF^b^ autosomal mCA	1.94	7.30 × 10^−2^	215	7,691
Lymphoid	ChrX mCA	1.23	2.96 × 10^−1^	215	7,691
Lymphoid	Autosomal L-mCA	5.64	6.60 × 10^−9^	215	7,691
Myeloid	Autosomal mCA	5.42	1.35 × 10^−5^	52	7,691
Myeloid	High CF^a^ autosomal mCA	7.77	1.74 × 10^−6^	52	7,691
Myeloid	Low CF^b^ autosomal mCA	2.61	1.92 × 10^−1^	52	7,691
Myeloid	ChrX mCA	1.08	8.56 × 10^−1^	52	7,691
MDS	Autosomal mCA	1.59	1.68 × 10^−1^	112	9,828
MDS	ChrX mCA	1.11	6.99 × 10^−1^	112	9,828

^a^High CF mCA was defined as having estimated CF ≥0.03.

^b^Low CF mCA was defined as having estimated CF <0.03.

MDS, myelodysplastic syndrome.

To investigate the broader impact that inflammatory and behavioral risk factors for blood cancers may have on mCAs, we ran association tests between mCAs and 19 blood cell traits, body mass index (BMI), C-reactive protein (CRP) levels, interleukin-6 (IL6) levels and smoking status (Methods) in up to 49,353 individuals. Of the 19 blood cell traits, we observed significant associations between mCA carrier status and levels of lymphocytes, neutrophils and total number of white blood cells (Table 3). Specifically, autosomal mCAs were associated with an increase in both lymphocyte counts (*β* = 0.025) and total white cell counts (*β* = 0.018); the associations were stronger when we considered mCAs at high CF. ChrX mCA status was associated with a decrease in neutrophil counts (*β* = −0.021) and percentages (*β* = −0.025), even after adjusting for potential confounding by the Duffy null variant, but was associated with an increase in lymphocyte counts (*β* = 0.030) and percentages (*β* = 0.022) (Table 3). We did not observe statistically significant associations between autosomal mCAs or chrX mCAs with smoking status, levels of CRP or levels of IL6 (Supplementary Table 18), although the estimated effect of smoking on presence of autosomal mCAs (OR 1.67) was similar to that from a previous study^24^. Finally, we observed that the presence of autosomal mCAs was associated with a significant decrease in BMI (*β* = −0.015, *P* = 0.002), although we were not able to determine the causal direction of this effect.

**Table 3:** Associations between quantitative blood cell counts and mCA phenotypes

Trait	Test	*β*	SE	*P* value	*n*
WBC	Autosomal	0.0180	0.00281	1.46 × 10^−10^	49,353
WBC	Autosomal, high CF Yes–No^a^	0.0266	0.00373	1.10 × 10^−12^	49,353
NEU	ChrX	−0.0215	0.00458	2.74 × 10^−6^	18,415
NEU	ChrX, adjusted for Duffy	−0.0155	0.00442	4.47 × 10^−4^	18,415
NEU%	ChrX	−0.0246	0.00314	5.12 × 10^−15^	16,248
NEU%	ChrX, adjusted for Duffy	−0.0242	0.00300	8.87 × 10^−16^	16,248
LYM	Autosomal	0.0246	0.00467	1.41 × 10^−7^	33,927
LYM	ChrX	0.0301	0.00371	5.81 × 10^−16^	19,658
LYM	Autosomal, high CF Yes–No^a^	0.0359	0.00638	1.77 × 10^−8^	33,927
LYM%	ChrX	0.0222	0.00243	8.11 × 10^−20^	17,245

^a^High CF mCA was defined as having estimated CF ≥0.03.

CF, clonal fraction; LYM, lymphocytes; NEU, neutrophils; SE, standard error; WBC, white blood cell counts.

## Discussion

In this study, we profiled the mCA landscape across an ancestrally diverse set of samples with WGS data to investigate the genomic distribution of mCAs and their germline genetic drivers. We observed differences in rates of mCAs across ancestry groups, confirming previous reports of higher prevalence of autosomal mCAs in individuals of EA relative to AA and EAS ancestry populations^16,25^ and found a lower rate of autosomal mCAs in HA ancestry individuals compared with EA individuals. For the first time, we showed that both AA and HA populations have higher rates of chrX mCAs compared with EA. Importantly, these cross-ancestry comparisons were confirmed with a robust downsampling procedure that removed potential confounding due to differential rates of heterozygosity across ancestries. We observed that autosomal mCAs rates across ancestry groups follow similar patterns observed for the incidence of leukemia across racial and ethnic groups as defined in the Surveillance, Epidemiology, and End Results database (https://seer.cancer.gov). Although ancestry is different from race and ethnicity (‘race’ and ‘ethnicity’ refer to non-biological social categories and ‘ancestry’ refers to genetic origins), our findings support the use of autosomal mCAs as an intermediate phenotype to study environmental and genetic drivers of blood cancer and as a biomarker for risk.

Both our study and previous studies of European and Japanese populations have uncovered germline variants that increase risk of mCAs, both in *cis* and in *trans* state^5,16^. In our cohort, we replicated previous associations at the SNP and gene level, demonstrating that rare variants from HA and AA populations are also associated with mCAs. Although some of these variants are ancestry specific, they share molecular drivers, for example with rare variants of large effects driving *cis* association of mCAs spanning *MPL* and *ATM*. An association between a germline variant with presence of mCAs may lend support for classification of a variant as pathogenic in patients with blood cancer, particularly in populations that are underrepresented in genetic variant databases.

Relative to what is observed in EA, rates of heterozygosity are higher in the HA and AA admixed populations^26^. Although this difference presents an advantage for detection of mCAs at lower cell fractions in AA and HA admixed populations, we demonstrate the importance of taking this into account when comparing mCA rates across individuals of different ancestries. From our analyses, this difference in sensitivity was most impactful in detection of chrX mCAs, which, relative to autosomal mCAs, were present at lower cell fractions. The high rates of chrX mCAs (particularly chrX losses) but at overall lower cell fractions suggests that clones with chrX mCAs may arise due to relatively weak positive selection of clones with these mutations and/or possibly high rates of chrX missegregation during cell divisions. Both of these explanations are supported by recent work that has shown a decrease in hematopoietic clonal diversity in elderly individuals (>75 years) and clonal expansions detectable through single-cell sequencing starting before the age of 40 (ref. ^27^). The observation of chrX mCAs at overall lower cell fractions and higher rates than autosomal mCAs suggests that autosomal mCAs may be under stronger positive selection relative to chrX mCAs.

Our study was the first of its kind to implement haplotype-based mCA detection methods on large-scale WGS data from a population-based cohort. With recent enhancement to the MOsaic CHromosomal Alterations (MoChA) mCA calling software, we were able to detect mCAs with lower CFs compared with array-based datasets. Our pipeline relied on substantial post hoc filtering of mCA calls. In particular, we discarded many small mCA calls due to our inability to distinguish gains, loss and CN-LOH events for mCAs <1 Mb in size. This decrease in sensitivity for small mCAs may be possible to overcome with higher coverage or improvements in the detection methods.

This work represents a large-scale effort to understand the co-occurrence of distinct forms of mosaicism in individuals of diverse ancestries. Prior work characterizing CHIP and mCAs has focused on individuals of East Asian ancestry, white British individuals or among individuals with solid tumors^23,28,29^. Similar to these efforts, we found that CN-LOH co-occurring with CHIP mutations is a common mechanism through which *TET2* (4q), *DNMT3A* (2p), *JAK2* (9p), *CUX1* (7q) and *TP53* (17p) acquire a competitive advantage. We also note that loss of 13q, a common alteration in CLL, co-occurred with CHIP mutations in *TET2* and *CBL*, which may explain how CHIP mutations, despite leading to a myeloid bias, may also predispose individuals to lymphoid malignancy through co-occurring mutations. The observation that *DNMT3A* mutations did not show co-occurrence with *cis*-mCAs at higher mutant cell fractions supports the findings by Uddien et al. and Fabre et al.^30,31^, showing that *DNMT3A* mutant clones exhibit slower growth rates compared with clones harboring other CHIP mutations. We also replicate some of the co-occurence patterns reported by Saiki et al.^28^ and report additional ones. Saiki et al. used targeted sequencing for SNV calling of CHIP mutations and array data for mCA detection participants in the BioBank Japan cohort. In contrast, we studied a diverse set of individuals living in the United States with WGS data. The differences in demographic factors and sensitivity for detection of CHIP/mCA mutations limit our ability to directly compare results between these two studies.

An important area of further investigation will focus on the distinction between heterozygosity and homozygosity at a CHIP locus and disease consequences. It is tempting to speculate that patients with clones that make up the same fraction of blood that are homozygous for CHIP mutations due to concomitant CN-LOH may have worse prognosis than individuals with a single mutation.

## Methods

### Study population

We included 67,390 participants from 19 TOPMed studies: Genetics of Cardiometabolic Health in the Amish (*n* = 1,109) (ref. ^32^), Atherosclerosis Risk in Communities Study (*n* = 3,780) (ref. ^33^), Barbados Genetics Asthma Study (*n* = 980), Mount Sinai BioMe Biobank (*n* = 9,392) (ref. ^34^), Coronary Artery Risk Development in Young Adults (*n* = 3,293) (ref. ^35^), Cleveland Family Study (*n* = 1,281), CHS (*n* = 3,517) (ref. ^36^), COPDGene (*n* = 10,050) (ref. ^37^), Framingham Heart Study (*n* = 4,007) (ref. ^38^), Genetic Studies of Atherosclerosis Risk (*n* = 1,733) (ref. ^39^), Genetic Epidemiology Network of Arteriopathy (*n* = 1,157), Genetics of Lipid Lowering Drugs and Diet Network (*n* = 942), Hispanic Community Health Study—Study of Latinos (*n* = 3,857) (ref. ^40^), Hypertension Genetic Epidemiology Network (*n* = 1,865), Jackson Heart Study (*n* = 3,317) (ref. ^41^), MESA (n = 5,222) (ref. ^42^), Vanderbilt BioVU study of African Americans (*n* = 1,085), Women’s Genome Health Study (*n* = 108), and Women’s Health Initiative (WHI, *n* = 10,695) (ref. ^43^). The 67,390 TOPMed participants were categorized into discrete ancestry subgroups using the Harmonized Ancestry and Race/Ethnicity machine learning algorithm^44^, which uses genetically inferred ancestry to refine self-identified race/ethnicity and impute missing racial/ethnic values. The ancestry composition in this study was 57% European, 30% African, 11% Hispanic/Latino and 2% Asian (Supplementary Table 19). Further descriptions of the design of the participating TOPMed cohorts and the sampling of individuals within each cohort for TOPMed WGS are provided in Supplementary Note 3. All studies were approved by the appropriate institutional review boards (IRBs) and informed consent was obtained from all participants.

### WGS data

WGS was performed as part of the National Heart, Lung and Blood Institute TOPMed program. The WGS was performed at an average depth of 38X by six sequencing centers (Broad Genomics, Northwest Genome Institute, Illumina, New York Genome Center, Baylor, and McDonnell Genome Institute) using Illumina X10 technology and DNA from blood. Here we report analyses from ‘Freeze 8’, for which reads were aligned to the Genome Reference Consortium human genome build 38 using a common pipeline across all centers. To perform variant quality control (QC), a support vector machine classifier was trained on known variant sites (positive labels) and Mendelian inconsistent variants (negative labels). Further variant filtering was done for variants with excess heterozygosity and Mendelian discordance. Sample QC measures included: concordance between annotated and inferred genetic sex, concordance between prior array genotype data and TOPMed WGS data, and pedigree checks. Additional details can be found in Taliun et al.^26^.

### Detection of mCAs

Detection of mCAs was performed on the WGS-based genotype and read depth data. The mCA call set was generated using the MoChA v1.11 caller. This approach utilizes phased genotypes, coverage (log R ratio, LRR) and B allele frequency (BAF) at heterozygous sites for detection of mCAs. Input data at heterozygous markers came from a previous analysis of the TOPMed cohort as outlined in Taliun et al.^26^. However, not all variants were included in the analyses. First, heterozygous markers with a MAF less than 1% and those where the read depth of either allele was less than five were removed. Second, we removed markers within germline copy number variants previously generated in TOPMed. Third, when more than one marker was present in a 1,000 base pair genomic region, then only one marker was retained. The MoChA caller was run with the extra option ‘–LRR-weight 0.0–bdev-LRR-BAF 6.0’ to disable the LRR + BAF model. The resulting mCA calls were filtered by excluding (1) those that span less than 2,000 informative markers, that is heterozygous sites; (2) those with logarithm of the odds score less than 5; (3) those on chrX but with inferred sex ‘unknown’; (4) those with estimated relative coverage higher than 2.9; and (5) those with BAF deviation larger than 0.16 and relative coverage higher than 2.5. Steps 4 and 5 are used to exclude putative germline duplications. Classification of mCAs as lymphoid or myeloid was performed following criterion from Niroula et al.^23^.

### Downsampling

The total number of heterozygous sites can affect the power for detection of mCAs as the mCA calling method relies on heterozygous sites for detecting imbalances in the parental haplotypes^5,45^. The AA, HA and EA groups had on average higher number of heterozygous sites compared with the EAS group (Supplementary Table 4); therefore, to adjust for this difference we downsampled heterozygous sites in the AA, HA and EA groups, and then used those data to generate mCA calls and reasses reported associations of mCAs with ancestry. The downsampling was conducted by matching the distribution of heterozygous sites for AA, HA and EA groups to that of the EAS group. This adjustment was done separately for females and males. For example, if a HA female sample had 925,935 heterozygous markers, which is equivalent to the 50th percentile for HA females, then heterozygous markers were removed at random across the genome until the sample had 749,959 markers, which is equal to the 50th percentile for EAS females.

### Comparisons of mCAs across ancestries

Subsequent to downsampling, we investigated possible batch effects that may have influenced mCA detection rates across both autosomes and chrX. After adjusting for age, age^2^, sex and ancestry, a variable representing ‘study’ had no effect on autosomal mCA detection, although we did find a study effect for chrX mCA detection. Therefore, in all of our analyses comparing mCA detection across ancestries, we included age, age^2^, sex and study as covariates.

### Estimation of genetic ancestry proportions

Ancestry was estimated in the TOPMed WGS data using RFMix^46^ with a three-way reference panel of 92 Europeans and 92 Africans from the 1,000 Genomes project^47^ and 92 Native American samples from Human Genome Diversity Project^48^. In TOPMed we only considered SNPs with MAF >0.05 to speed up the computation. RFMix was run separately for each chromosome. We estimated the proportion of African ancestry for all AA individuals at the chromosome level by averaging the local ancestry proportions of all loci within that chromosome. To determine whether estimated African ancestry on chrX was associated with the prevalence of an mCA on that same chromosome, we ran logistic regressions with mCA status as the response variable, age, age^2^, sex and study as covariates, and the four quartiles of estimated African ancestry as the main effect of interest, with 0–25% as the reference group. For autosomal mCAs (Supplementary Table 9) African ancestry was treated as a continuous variable.

### Association analyses

We performed a WGS-based GWAS between germline variants observed in TOPMed and presence of an mCA, separately for autosomal (*N* = 67,518) and chrX loss (*N* = 39,585) mCAs. For each sample, we defined the phenotype as presence/absence of one or more autosomal mCAs and tested against all variants with MAC ≥ 5 that passed the quality filters. Samples with uncertain identity or poor quality were excluded from analysis. For chrX loss analyses we excluded samples with a chrX mCA that was a gain, CN-LOH or undetermined. Principal components and genetic relatedness estimates were calculated using PC-AiR^49^ and PC-Relate^50^, as described previously in Hu et al.^51^ QC replicates or duplicate samples were removed after selecting the sample with the highest average autosomal depth rate. All logistic regression analyses included age, age^2^, sex, study and genetic ancestry as covariates. The final sample set included five genetic ancestry categories consisting of AA, EA, EAS, HA and a group of 1,099 samples that were characterized as having “unknown” ancestry. To test for association of sex with specific mCA types, for example 20q loss, we first conducted a chi-squared test (R chisq.test, simulate.p.value = TRUE, *B* = 100,000). For mCA types with marginal significance (*P* < 0.1), we then conducted logistic regression to test for association using a Bonferroni correction to account for the 156 independent tests.

We performed genetic association tests in *cis* and in *trans* state using a generalized linear mixed model approach using the generalized linear mixed model association test method^52^ as implemented in the GENESIS software^53^. For each analysis, a null model assuming no association between the outcome and any variant was fit, adjusting for sex, age, study-sequencing phase and the first 11 principal components (PCs) to capture genetic ancestry. A fourth degree sparse empirical kinship matrix computed with PC-Relate was included as a random effect to account for genetic relatedness among participants. The residuals from this null model were then used to perform genome-wide score tests of genetic association.

For the *trans* association analyses, we defined cases as those with a detectable mCA and tested all genetic variants with MAC ≥20 and had less than 10% of samples with sequencing read depth <10 at that particular variant.

For the *cis* associations (that is, variants within the same genomic locus as the mCA), we identified eight genomic loci of interest, which included the *ATM* and *MPL* genes, as well as chromosome arms with recurrent autosomal mCAs (*n* > 100), which included 14q CN-LOH, 1q CN-LOH, 11q CN-LOH, 12p gain, 12q gain, 20q loss and 1q CN-LOH. Cases were defined as those with an mCA call spanning the chromosome arm or gene, while controls were defined as those without any mCA calls on the chromosome arm tested. Because the case-control ratio was highly unbalanced for these analyses, we matched cases to controls using study, sequencing phase, sex and age to obtain a 1:10 ratio before fitting the null model. We tested all variants that passed the quality filters and had MAF ≥0.01. We defined a significance threshold of *P* < 0.05/(effective number of variants), where the effective number of variants tested was calculated using simpleM^54^.

For the *ATM* and *MPL* gene analyses, we further filtered variants based on annotations. Variants were annotated using ANNOVAR (v2019-10-24) and selected for use on the basis of their presence in exons and/or potential involvement in splicing. In addition to canonical splice sites, we also tested variants ±6 bp from exon boundaries, as well as less-canonical splice sites identified by SPIDEX^47,55^. To specifically account for promoters, we identified promoters for these two genes using the Eukaryotic Promoter Database and included variants found in the promoter region. Similar to the *cis*-association analyses, we defined a significance threshold of *P* < 0.05/(effective number of variants), where the effective number of variants tested was calculated using simpleM. We also ran secondary analyses of these variants with a lower MAC threshold (MAC ≥5).

In addition to single variant testing, we conducted gene-based aggregate tests to assess the cumulative effect of rare variants on mCA presence. Variants were aggregated by gene using the GENCODE v29 gene model. We used two strategies for filtering variants. For both strategies, variants were first filtered to MAF <0.01 in the sample set being tested. The first strategy (coding_filter1) includes only high confidence predicted loss-of-function variants inferred using LOFTEE^56^ and missense variants filtered using MetaSV score >0 (ref. ^57^). The second strategy (coding_noncoding_filter1) includes all variants from the first strategy plus additional regulatory variants. Regulatory variants were included if they overlapped with enhancer(s) or promoters linked to a gene using GeneHancer^58^ or 5 Kb upstream of the transcription start site. Within these regions only those variants were retained that had Fathmm-XF score >0.5 or overlap with regions labeled as either ‘CTCF binding sites’ or ‘transcription factor binding sites’ as annotated by the Ensembl regulatory build annotation^59^. Results were filtered to only those aggregation units with a cumulative MAC ≥20. We defined the significance threshold as *P* < 0.05/(number of aggregation units tested).

The annotation-based variant filtering and gene-based aggregation was performed using TOPMed Freeze 8 Whole Genome Sequence Annotator (WGSA) Google BigQuery annotation database on the BiodataCatalyst powered by Seven Bridges platform^60^. The annotation database was built using variant annotations generated by WGSA version v0.8 (ref. ^61^) and formatted by WGSAParsr version 6.3.8 (ref. ^62^). The GENCODE v29 gene model-based variant consequences were obtained from Ensembl Variant Effect Predictor^63^ incorporated within WGSA.

### Allelic shift analysis

Allelic shift analyses were conducted as outlined in Loh et al.^6^. Variants in the *MPL* and *ATM* genes with *P* < 0.05 in the *cis*-association analyses and with OR >1 were included in these analyses.

### Co-occurence analysis

Co-occurence between CHIP status and mCA status was analyzed as in previous work^28^. First, CHIP ‘carriers’ (individuals with an observed acquired mutation) were assigned to different categories by the gene where the mutations were located. Carriers of mCAs were assigned to categories based on the location (chromosome, p-arm and q-arm) and the changes of copy numbers (gain, loss and CN-LOH) of the mCA. A CHIP carrier or an mCA carrier may have been assigned to different categories if that individual carried multiple CHIP mutations or multiple mCAs. In our analysis, we required there to be at least ten carriers in each category, leaving 30 CHIP categories and 66 mCA categories for comparison (Fig. 3b). *P* values for co-occurence of CHIP and mCA carrier status were obtained via the Wald test (note that 0.5 was added to all cells in the 2 × 2 table if zero value(s) exist). A Bonferroni correction was implemented to assess significance.

### Analysis of hematologic malignancy data

Due to a paucity of cancer outcome data in all other cohorts, we restricted our analysis of hematologic malignancies to the WHI. We assigned the available hematologic cancer outcomes in the WHI cohort into categories: lymphoid and myeloid cancers. Patients diagnosed as chronic lymphocytic leukemia, non-Hodgkins lymphoma or multiple myeloma were assigned to the lymphoid group (*n* = 237). All other patients diagnosed with leukemia were assigned to the myeloid group (*n* = 53). We further excluded patients who were diagnosed as having any cancer before blood draw (that is, the time at which DNA for mCA calling was collected), which reduced the lymphoid cancer case number to 223 and the myeloid cancer case number to 52.

We ran separate logistic regression models to test the association between mCA carrier status and risk for lymphoid or myeloid cancer. Covariates in our model included CHIP carrier status, the interaction between CHIP carrier status and mCA carrier status, age, ancestry and smoking. Ancestry groups with fewer than five cases were not included in this analysis. To determine whether any associations with myeloid or lymphoid malignancies were implicating mCAs as biomarkers for early subclinical disease, we re-ran these logistic regressions excluding individuals with cytopenias or cytoses. Cytopenia and cytosis cases were defined on the basis of the blood cell counts collected at any of three WHI visits, using the definitions in Supplementary Table 20.

### Analysis with inflammatory and blood cell traits

A description of the measurement and quality control of the blood cell and inflammation traits can be found in Stilp et al.^64^. Each trait was defined as follows: hematocrit is the percentage of volume of blood that is composed of red blood cells. Hemoglobin is the mass per volume (g dl^−1^) of hemoglobin in the blood. Mean corpuscular hemoglobin is the average mass in picograms (pg) of hemoglobin per red blood cell. Mean corpuscular hemoglobin concentration is the average mass concentration (g dl^−1^) of hemoglobin per red blood cell. Mean corpuscular volume is the average volume of red blood cells, measured in femtoliters (fl). Red blood cell count is the count of red blood cells in the blood, by number concentration in millions per microliter (µl). Red cell distribution width is the measurement of the ratio of variation in width to the mean width of the red blood cell volume distribution curve taken at ±1 coefficient of variation. Total white blood cell count (WBC), neutrophil (NEU), monocyte, lymphocyte (LYM), eosinophil, basophil (BASO) and platelet count are defined with respect to cell concentration in blood, measured in thousands per microliter (µl). Because of a typical large point mass at zero, we dichotomized the BASO phenotype at BASO >0. The proportion of neutrophils, monocytes, lymphocytes or eosinophils was calculated by dividing the respective WBC subtype count by the total measured WBC. Mean platelet volume was measured in femtoliters. CRP was measured in mg l^−1^. IL6 was measured in pg ml^−1^. BMI was calculated from standing height and weight and smoking was dichotomized as ever/never smoker.

We tested for the association between each of these traits and presence of autosomal mCAs, chrX mCAs and mCAs at either high (>3%) or low CF. We ran standard linear models treating each quantitative trait as the dependent variable and presence/absence of mCA as the main independent variable of interest, while adjusting for age, sex, ancestry group and TOPMed study phase. BMI, WBC, NEU, LYM, monocytes and eosinophils were log transformed. We ran logistic regressions to test the association with smoking and BASO, adjusting for the same set of covariates. R version 4.2.1 was used for all statistical analyses.

### Statistics and reproducibility

No statistical methods were used to predetermine sample size. There were no interventions to which subjects were randomized. The mCA calls were filtered by excluding (1) those that span less than 2,000 informative markers, that is, heterozygous sites; (2) those with logarithm of the odds score less than 5; (3) those on chrX but with inferred sex ‘unknown’; (4) those with estimated relative coverage higher than 2.9; and (5) those with BAF deviation larger than 0.16 and relative coverage higher than 2.5. Steps 4 and 5 are used to exclude putative germline duplications. Step 3 was used to exclude potential sex mismatches and steps 1 and 2 were used to exclude low confidence mCAs. For the associations with hematologic malignancies, we excluded patients who were diagnosed as having any cancer before blood draw. To assess the possibility that the associations with hematologic malignancies were implicating mCAs as biomarkers for early subclinical disease, we re-ran the associations excluding individuals with cytopenias or cytoses as defined in Supplementary Table 20. Samples with uncertain identity or poor quality were excluded from germline association analyses. For chrX loss analyses we excluded samples with a chrX mCA that was a gain, CN-LOH or undetermined.

### Reporting summary

Further information on research design is available in the Nature Portfolio Reporting Summary linked to this article.

## Online content

Any methods, additional references, Nature Portfolio reporting summaries, source data, extended data, supplementary information, acknowledgements and peer review information; details of author contributions and competing interests; and statements of data and code availability are available at 10.1038/s41588-023-01553-1.

### Supplementary information

Supplementary Information Supplementary note and methods.

Reporting Summary

Supplementary Tables Supplementary Tables 1–21.

Supplementary Data Burden plots of Cholesky residuals for each variant included in the aggregate rare-variant tests for the associations in Table 1.

## Data Availability

Data for each participating study can be accessed through dbGaP with the corresponding TOPMed accession numbers: Amish (phs000956), ARIC (phs001211), BioMe (phs001644), BAGS (phs001143), CARDIA (phs001612), CFS (phs000954), CHS (phs001368), COPDGene (phs000951), FHS (phs000974), GeneSTAR (phs001218), GENOA (phs001345), GOLDN (phs001359), HCHS/SOL (phs001395), HyperGEN (phs001293), JHS (phs000964), MESA (phs001416), VU_AF (phs001032), WGHS (phs001040) and WHI (phs001237). We also accessed the Eukaryotic Promoter Database (https://epd.expasy.org/epd) for variant annotation.

## Code availability

Code to implement the mCA calling is available on GitHub at https://github.com/auerlab, which has scripts used for input data filtering, and https://github.com/freeseek/mocha with scripts for mCA detection and filtering.
