# Supplementary material for: Mosaic chromosomal alterations in blood across ancestries using whole-genome sequencing
Source: Nat Genet. 2023 Oct 30;55(11):1912–9. doi: 10.1038/s41588-023-01553-1 (PMC10632132; doi:10.1038/s41588-023-01553-1)
Supplement: Supplementary file 2 — Reporting Summary [file 41588_2023_1553_MOESM2_ESM.pdf]

nature portfolio

Corresponding author(s): Auer and Scheet

Last updated by author(s): September 12, 2023

# Reporting Summary

Nature Portfolio wishes to improve the reproducibility of the work that we publish. This form provides structure for consistency and transparency in reporting. For further information on Nature Portfolio policies, see our Editorial Policies and the Editorial Policy Checklist.

## Statistics

For all statistical analyses, confirm that the following items are present in the figure legend, table legend, main text, or Methods section.

| n/a | Confirmed | |
|---|---|---|
| ☐ | ☒ | The exact sample size (*n*) for each experimental group/condition, given as a discrete number and unit of measurement |
| ☐ | ☒ | A statement on whether measurements were taken from distinct samples or whether the same sample was measured repeatedly |
| ☐ | ☒ | The statistical test(s) used AND whether they are one- or two-sided<br>*Only common tests should be described solely by name; describe more complex techniques in the Methods section.* |
| ☐ | ☒ | A description of all covariates tested |
| ☐ | ☒ | A description of any assumptions or corrections, such as tests of normality and adjustment for multiple comparisons |
| ☐ | ☒ | A full description of the statistical parameters including central tendency (e.g. means) or other basic estimates (e.g. regression coefficient) AND variation (e.g. standard deviation) or associated estimates of uncertainty (e.g. confidence intervals) |
| ☐ | ☒ | For null hypothesis testing, the test statistic (e.g. $F$, $t$, $r$) with confidence intervals, effect sizes, degrees of freedom and $P$ value noted<br>*Give P values as exact values whenever suitable.* |
| ☒ | ☐ | For Bayesian analysis, information on the choice of priors and Markov chain Monte Carlo settings |
| ☒ | ☐ | For hierarchical and complex designs, identification of the appropriate level for tests and full reporting of outcomes |
| ☒ | ☐ | Estimates of effect sizes (e.g. Cohen's *d*, Pearson's *r*), indicating how they were calculated |

*Our web collection on statistics for biologists contains articles on many of the points above.*

## Software and code

Policy information about availability of computer code

| | |
|---|---|
| Data collection | No software was used to collect the data in this study. |
| Data analysis | Analysis scripts can be found at https://github.com/auerlab. R version 4.2.1 was used for statistical analyses. The GENESIS R-package was also used for the genetic association analyses. |

For manuscripts utilizing custom algorithms or software that are central to the research but not yet described in published literature, software must be made available to editors and reviewers. We strongly encourage code deposition in a community repository (e.g. GitHub). See the Nature Portfolio guidelines for submitting code & software for further information.

## Data

Policy information about availability of data

All manuscripts must include a data availability statement. This statement should provide the following information, where applicable:

- Accession codes, unique identifiers, or web links for publicly available datasets
- A description of any restrictions on data availability
- For clinical datasets or third party data, please ensure that the statement adheres to our policy

Data for each participating study can be accessed through dbGaP with the corresponding TOPMed accession numbers: Amish (phs000956), ARIC (phs001211), BioMe (phs001644), BAGS (phs001143), CARDIA (phs001612), CFS (phs000954), CHS (phs001368), COPDGene (phs000951), FHS (phs000974), GeneSTAR

(phs001218), GENOA (phs001345), GOLDN (phs001359), HCHS/SOL (phs001395), JHS (phs000964), MESA (phs001416), VU_AF (phs001032), WGHS (phs001040), WHI (phs001237). We also accessed the Eukaryotic Promoter Database (https://epd.expasy.org/epd) for variant annotation.

## Human research participants

Policy information about studies involving human research participants and Sex and Gender in Research.

| | |
|---|---|
| Reporting on sex and gender | Sex was determined based on self-report. |
| Population characteristics | Covariate relevant population characteristics are reported in the Supplement. |
| Recruitment | Details of participant recruitment can be found in the Supplement. |
| Ethics oversight | All studies were approved by the appropriate institutional review boards (IRBs) and informed consent was obtained from all participants. |

Note that full information on the approval of the study protocol must also be provided in the manuscript.

# Field-specific reporting

Please select the one below that is the best fit for your research. If you are not sure, read the appropriate sections before making your selection.

☒ Life sciences ☐ Behavioural & social sciences ☐ Ecological, evolutionary & environmental sciences

For a reference copy of the document with all sections, see nature.com/documents/nr-reporting-summary-flat.pdf

# Life sciences study design

All studies must disclose on these points even when the disclosure is negative.

| | |
|---|---|
| Sample size | Sample size was determined as the largest set of samples with whole-genome sequence data for which mCA calling could be performed. |
| Data exclusions | None. |
| Replication | We conducted a study to replicate our mCA calls using array-based data from the WHI, CHS, MESA, and COPDGene studies. |
| Randomization | There was no randomization in our study because there was no intervention with subjects. |
| Blinding | Blinding was not relevant because there was no intervention with subjects. |

# Reporting for specific materials, systems and methods

We require information from authors about some types of materials, experimental systems and methods used in many studies. Here, indicate whether each material, system or method listed is relevant to your study. If you are not sure if a list item applies to your research, read the appropriate section before selecting a response.

## Materials & experimental systems

| n/a | Involved in the study |
|---|---|
| ☒ | ☐ Antibodies |
| ☒ | ☐ Eukaryotic cell lines |
| ☒ | ☐ Palaeontology and archaeology |
| ☒ | ☐ Animals and other organisms |
| ☒ | ☐ Clinical data |
| ☒ | ☐ Dual use research of concern |

## Methods

| n/a | Involved in the study |
|---|---|
| ☒ | ☐ ChIP-seq |
| ☒ | ☐ Flow cytometry |
| ☒ | ☐ MRI-based neuroimaging |
